# A Virtual 3D Dynamic Model of Caries Lesion Progression as a Learning Object for Caries Detection Training and Teaching: Video Development Study

**DOI:** 10.2196/14140

**Published:** 2020-05-22

**Authors:** Juan Sebastian Lara, Mariana Minatel Braga, Carlos Gustavo Zagatto, Chao Lung Wen, Fausto Medeiros Mendes, Pedroza Uribe Murisi, Ana Estela Haddad

**Affiliations:** 1 Department of Cariology, Operative Dentistry and Dental Public Health Indiana University School of Dentistry Indianapolis, IN United States; 2 Department of Pediatric Dentistry School of Dentistry University of Sao Paulo Sao Paulo Brazil; 3 Discipline of Telemedicine, Department of Pathology School of Medicine University of Sao Paulo Sao Paulo Brazil; 4 Department of Pediatric Dentistry Dental School University of Guadalajara Guadalajara Mexico

**Keywords:** 3d virtual models, dental education, e-learning, learning object, caries, cariology

## Abstract

**Background:**

In the last decade, 3D virtual models have been used for educational purposes in the health sciences, specifically for teaching human anatomy and pathology. These models provide an opportunity to didactically visualize key spatial relations that can be poorly understood when taught by traditional educational approaches. Caries lesion detection is a crucial process in dentistry that has been reported to be difficult to learn. One especially difficult aspect is linking clinical characteristics of the different severity stages with their histological features, which is fundamental for treatment decision-making.

**Objective:**

This project was designed to develop a virtual 3D digital model of caries lesion formation and progression to aid the detection of lesions at different severity stages as a potential complement to traditional lectures.

**Methods:**

Pedagogical planning, including identification of objectives, exploration of the degree of difficulty of caries diagnosis–associated topics perceived by dental students and lecturers, review of the literature regarding key concepts, and consultation of experts, was performed prior to constructing the model. An educational script strategy was created based on the topics to be addressed (dental tissues, biofilm stagnation areas, the demineralization process, caries lesion progression on occlusal surfaces, clinical characteristics related to different stages of caries progression, and histological correlations). Virtual 3D models were developed using the Virtual Man Project and refined using multiple 3D software applications. In the next phase, computer graphic modelling and previsualization were executed. After that, the video was revised and edited based on suggestions. Finally, explanatory subtitles were generated, the models were textured and rendered, and voiceovers in 3 languages were implemented.

**Results:**

We developed a 6-minute virtual 3D dynamic video in 3 languages (English, Spanish, and Brazilian Portuguese) intended for dentists and dental students to support teaching and learning of caries lesion detection. The videos were made available on YouTube; to date, they have received more than 100,000 views.

**Conclusions:**

Complementary pedagogical tools are valuable to support cariology education. This tool will be further tested in terms of utility and usability as well as user satisfaction in achieving the proposed objectives in specific contexts.

## Introduction

Dental caries is one of the most prevalent chronic diseases worldwide [1]. It is caused by the interaction of several factors that culminate in dissolution of the localized chemical tooth structure by metabolic events occurring in the oral biofilm [2]. This cumulative mineral loss is known as a caries lesion; these lesions can vary from simple changes in enamel translucency to extensive cavities involving the dentine and pulp [3].

Dental caries are detected by recognizing the signs and symptoms involved in the abovementioned process [4]. The importance of caries detection lies in the possibility of confirming the presence or absence of disease, assessing its prognosis, contributing to the decision-making process, informing the patient, and monitoring the clinical course of the disease [5]. In this sense, adequate caries detection is fundamental for planning and implementing health policies aimed to control the disease [6].

Several caries classification and detection methods have been developed to assess different stages of caries lesions [7]. However, the many differences and lack of standardization of these methods highlight the need to develop a defined, standardized, and validated caries detection system based upon current scientific evidence and the consensus of experts in the field of cariology [8]. In this regard, the International Caries Detection and Assessment System (ICDAS) [9] was designed to detect 6 stages of the caries process according to the disease severity, ranging from early visual changes in the enamel to extensive cavitation. Although this system has been widely used and has been shown to contribute to more accurate caries lesion detection [10], developing teaching tools is important and necessary to achieve and effectively disseminate new concepts and paradigms to facilitate their understanding and use [11]. These tools will reduce the difficulties of applying such concepts in a clinical scenario.

Based on this, the ICDAS Foundation designed an e-learning program to universalize and spread the use of their system [12]. This free 90-minute tool can be accessed online in 4 different languages to support training, provide dental examination protocols, and review the scoring system. Although the ICDAS e-learning program has been shown to improve the diagnostic skills of dental students for the detection of occlusal caries [13,14], specific clinical characteristics of different stages of caries lesion progression could not be linked to their respective histopathological features, which is important to understand the prognosis and influence of these stages on clinical decision-making.

**Figure 1:**
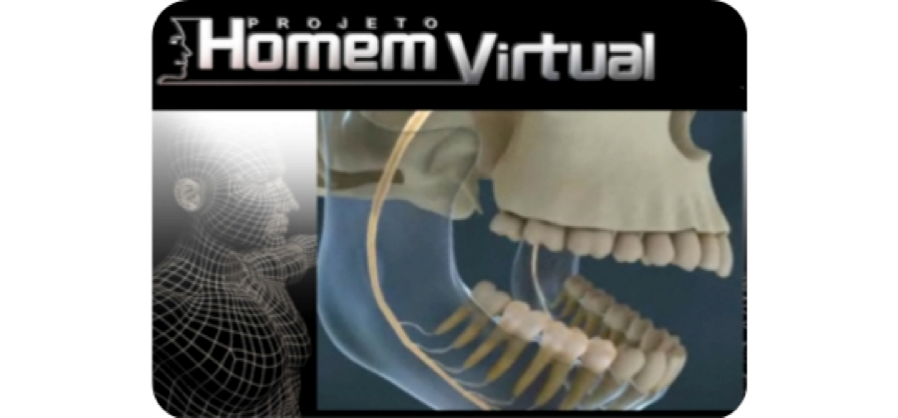
The Virtual Man Project developed at the Telemedicine Discipline, School of Medicine, University of Sao Paulo, Brazil.

3D animation models can show spatial and dynamical relationships from almost any angle; this can provide information that may be difficult to acquire using traditional static learning resources [15]. In this sense, the Virtual Man Project [16] developed at the Telemedicine Discipline of the University of Sao Paulo creates 3D images and animations of the human body that aid the comprehension of anatomy, physiology, pathologies, drug interactions, and surgical techniques in several areas (Figure 1).

Thus, the aim of the present project was to develop a digital, dynamic, and virtual 3D model of the formation, progression, and detection of caries lesions at different stages using the ICDAS at the Virtual Man Project Laboratory to complement traditional teaching resources.

## Methods

This descriptive study was developed in collaboration with the Discipline of Telemedicine (School of Medicine), the Teledentistry Centre, and the Department of Pediatric Dentistry (School of Dentistry) at the University of Sao Paulo, Brazil. The study was approved by the Ethics Committee of the Dental School (protocol 206.345/2013).

### Pedagogic Planning

This phase comprised the initial steps to develop a learning object oriented toward the detection of caries lesions based on their developmental stages and the differences among their clinical characteristics. In this phase, we discussed the objectives, the topics to approach, and the best methodologies for knowledge transmission. Firstly, a team of experienced lecturers and researchers in the area of cariology was formed to discuss the learning object purpose, key topics representing the minimum skills a dental student should develop in this field as a future dental practitioner, and the possibility of using technology to achieve the proposed goals. Sources such as the First Consensus Workshop on the Development of a European Curriculum in Cariology [17] and a study on current cariology education in dental schools in Spanish-speaking Latin American countries [18] were considered at this point.

As a second step in the previous study, we assessed the degrees of difficulty of caries detection–related learning topics perceived by dental students and lecturers [19]. In this phase, we used a conjoint analysis survey to determine the most difficult topics to learn regarding the detection of caries lesions [20]. In this survey, respondents were asked to rate the perceived degree of difficulty not by individual subactions but in combinations known as profiles. Conjoint analysis allows the identification of subactions that are considered to be better or worse examples of each research engagement action by calculating numerical weights, which are called utilities. These utilities represent the score to be assigned to each subaction. The topic considered to be the most difficult by students and lecturers was the histology of caries lesions (the correlation between the clinical characteristics of a caries lesion and its histological depth). Therefore, we based the construction of the present learning object on this specific subject to address this difficulty didactically.

### Graphic Design and Video Production

First, images of clinical and histological caries lesions were acquired to include them in the learning object as complementary supports. To that end, an examiner trained in the ICDAS criteria selected a sample of human teeth (N=12) with different caries lesion severity stages on occlusal surfaces (scores 0-6) from the Human Teeth Bank of the Dental School, University of Sao Paulo. Clinical images of these lesions were then obtained with a digital camera (DS126151 EOS Digital Rebel XTi, Canon) with a macro lens (EF 100mm 1:2.8, Canon). Then, the teeth were fixed with the crown exposed to Eppendorf tubes using utility wax and transparent acrylic resin. Longitudinal sections (100 micrometers) were made at the center of each lesion using a cutting machine (IsoMet 1000 precision sectioning saw, Buehler) with a diamond grinding disc (Extec 12205). The histological sections were analyzed under a stereomicroscope (M80, Leica), and the images were captured and processed with a digital camera (DFC 295, Leica) and QWin Plus software (Leica). This material was saved in digital files until its inclusion in the learning object.

A technological plan structure and interactive tele-education strategies were developed in partnership with a multidisciplinary team composed of cariology experts, digital designers, journalists, and tele-education strategists. This phase was carried out at the Virtual Man Project Laboratory using dual Pentium 4 graphic workstations (Xeon HT) with 4 gigabytes of RAM, a professional video board, tablets, the 3D Studio Max program (AutoDesk, Inc), and Photoshop and After Effects software (Adobe, Inc).

Before graphical production, a descriptive lecture regarding the caries process and the ICDAS criteria on occlusal surfaces was given to the team members who were not familiar with cariology. Concept descriptions as well as clinical and histological images of caries lesions were shown. Then, an educational script strategy was created to define the sequence in which the topics would appear in the video, emphasizing the most difficult topics (Textbox 1).

**Educational script showing the sequence of topics in the constructed video according to the pedagogical planning.**

**Sequence of topics**

1. Presentation
2. Dental structures (enamel, dentin, pulp)
3. Dental enamel structure, microscopic view
4. Dentine structure, microscopic view
5. Plaque stagnation areas
6. Demineralization process
7. Caries lesion formation
8. ICDAS scores (clinical/histological)
9. ICDAS scores (histological correlation)
10. Credits

**Figure 2:**
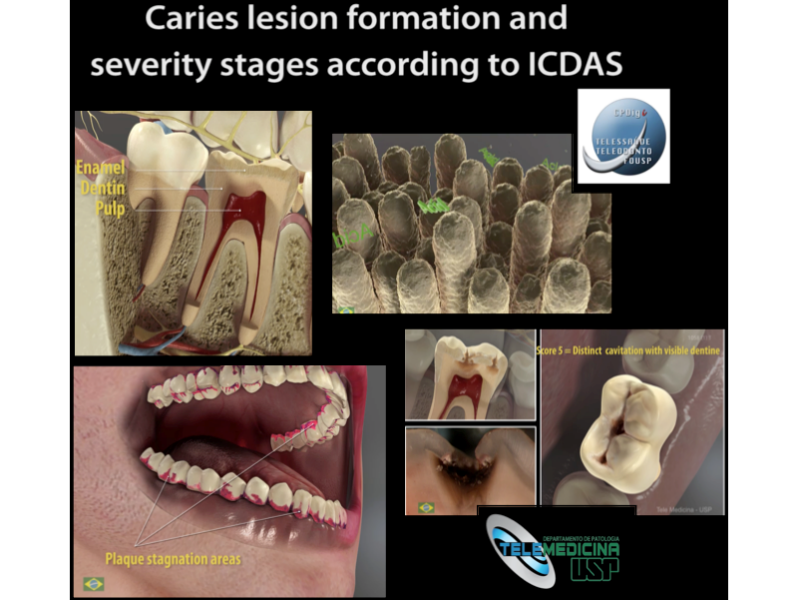
Scenes of the video produced at the Virtual Man Laboratory. ICDAS: International Caries Detection and Assessment System.

Graphical computer models (using the Virtual Man Project) of the clinical and histological images and a preview video were promptly generated. A group of experts in the field of cariology who were not involved in the video production reviewed this first version of the video. After that, the video was edited to correct some theoretical and technical inconsistencies. Then, a new version was produced that incorporated all the suggestions; after the team’s approval, this video was rendered and textured. To achieve this, an image synthesis process was performed to generate photorealistic images (geometry, viewpoint, texture, lighting, shading, etc.) from the developed 3D models using computer programs. By this process, a silent 6-minute video was created using the Virtual Man Project (Figure 2).

### Voiceover Recording

**Figure 3:**
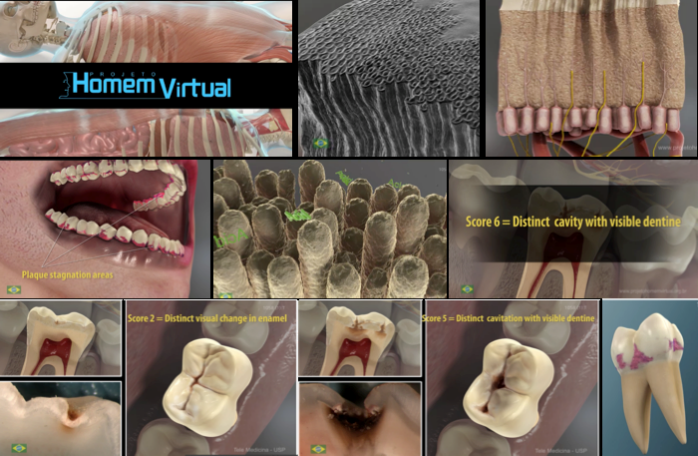
Frames from the final English version of the 6-minute video about the caries process and the International Caries Detection and Assessment System.

After the graphic design phase was complete, voiceovers were recorded to be played over the video to narrate the dynamic illustrations in an understandable way. Scripts in 3 languages (English, Spanish, and Brazilian Portuguese) were written and revised by native experts. After that, the scripts were adjusted and synchronized with the times and sequences in the video. We engaged a native-speaking narrator from the United Kingdom, Mexico, and Brazil. Each of the 3 narrators received a brief explanation about the video and its objectives together with the script. They were then taken into a recording studio and provided with instructions on how to record the voiceovers. Voice volume and speed tests were performed. Later, the narrator read the script while the voiceover was recorded. If the narrator made mistakes, they were required to start over from the previous paragraph. Repetitions were made when necessary. Finally, the recordings of the scripts were edited and incorporated into the body of the video. The final versions, a 6-minute video in each language, were sent to the scientific board for revision. Minimal corrections and additions were performed at this point (Figure 3).

## Results

Using the process described in the Methods section, a 6-minute dynamic video was produced in 3 languages (English, Spanish, and Brazilian Portuguese) showing the dental structures, biofilm stagnation areas, caries lesion formation, demineralization process, caries lesion progression, and severity stages of caries lesions on occlusal surfaces according to the ICDAS (Figure 2 and Figure 3). This process, from conception to the final product, required approximately 2 years. The first 6 months were dedicated to idealization of the project, formation of the team, and design of the methodology to assess the topics to be included in the learning object. In the following months, the multidisciplinary team was formed and the audiovisual production proceeded. Finalization of the audiovisual production required approximately 1.5 years. This included product conception, design, production, editing, rendering, and voiceover incorporation. The project leader was exclusively dedicated to the project, and the Virtual Man Project Laboratory staff worked an average of 20 hours per week on the production of the material. All 3 videos [21-23] were uploaded to YouTube in 2016; since then, they have received more than 130,000 views (English version: 33,000, Spanish version: 28,000, and Brazilian Portuguese version: 72,000). The 3 versions of the learning object can be accessed on the YouTube platform using the keywords “ICDAS” and “caries”.

## Discussion

### Principal Findings

Interest is increasing in developing educational resources using information and communication technology to improve students’ understanding of human body processes [15]. The developed tool is presented in an audiovisual media format that is compatible with computers, tablets, and smartphones using the Virtual Man Project as an innovative, dynamic, and directed communication method. The tool implements 3D graphical modeling and uses a visual classification system to transmit knowledge associated with caries lesion formation and its clinical manifestations based on the developmental stages of caries, which may benefit the caries detection process [10].

This project represents an improvement in educational infographics, as it may facilitate and accelerate understanding related to a specific matter [20]. The Virtual Man Project Laboratory at the University of Sao Paulo had previously developed some dentistry-related content, such as tooth extraction and mandibular nerve anesthesia [24] and atraumatic restorative treatment [25]. This learning object represents the continuous production of learning tools in the area of dentistry, specifically cariology. When watching the video, viewers can observe 3D animated anatomical structures that simulate the demineralization process due to bacterial acid production, the caries process, and the severity stages of lesions as well as their histological correlations; these are difficult topics to assimilate by conventional methods [15,26].

Part of the pedagogical planning for the development of this learning object was based on the findings of a previous study [19]. Those findings were extremely important, as they guided the development of the learning object based on students’ real expectations to stimulate ideal achievement of the knowledge and skills required to detect caries lesions in a clinical scenario. However, the impact that this learning object will have on students’ learning and competence acquisition is a matter of future study.

We consider that this tool may have an impact on the theoretical understanding of caries lesion formation and progression and therefore may improve students´ knowledge and grades. This is supported by a study in which students who accessed virtual tools scored higher on assessments than students who did not [27]. This tool is important because the implementation of multimedia designs for anatomical teaching purposes reduces students’ cognitive load [26] and permits dissemination of information to more students, with significant effects on improving their understanding of the relevant morphology [26].

As mentioned, the ICDAS e-learning program is an interactive resource that supports training in the use of the ICDAS criteria for dental education, examination protocols, and scoring systems [13] using static images, text, and voiceover recordings. The advantage of dynamically correlating the clinical process with the histopathological features of disease in the presented learning object may complement not only existing online resources but also traditional lectures, helping educators improve their teaching methodology [27].

One of our main goals is the dissemination of the produced tool. Open access is an ideal aspect of this process, and the first version of the video is already available on YouTube. However, additional steps should be performed to test the characteristics of the learning material as well as to address copyright issues; these steps are currently in progress. In the phase described in this paper, the authors created a learning object in 3 different languages: English, Spanish, and Brazilian Portuguese. This can be seen as an advantage in terms of dissemination, as these 3 languages have some of the largest populations of first-language speakers in the world [28].

As a next step to fully address the efficacy of the current learning object, the authors will test this tool in a student population in different contexts and countries. This testing will be conducted to validate and assess the potential benefits, obstacles, and user acceptability of the learning object as a novel pedagogical resource in the area of cariology. Therefore, we will perform a multicenter randomized study involving dental students from different countries with the aim of evaluating the impact of the 3D virtual model as a learning object on the training and teaching of undergraduate dental students to detect caries lesions using the ICDAS; the results of this study will be discussed in a future paper.

### Conclusions

We produced a 6-minute virtual 3D video intended for dentists and dental students to support teaching and learning of the caries detection process. We suggest that complementary pedagogical tools, such as the one described here, are valuable to complement education in cariology. This learning object will be further tested in terms of utility and usability as well as user satisfaction in achieving the proposed objectives in specific contexts.

## Abbreviations

ICDAS: International Caries Detection and Assessment System
